# Histone Deacetylases (HDAC) Inhibitor—Valproic Acid Sensitizes Human Melanoma Cells to Dacarbazine and PARP Inhibitor

**DOI:** 10.3390/genes14061295

**Published:** 2023-06-20

**Authors:** Małgorzata Drzewiecka, Anna Gajos-Michniewicz, Grażyna Hoser, Dominika Jaśniak, Gabriela Barszczewska-Pietraszek, Przemysław Sitarek, Piotr Czarny, Janusz Piekarski, Maciej Radek, Małgorzata Czyż, Tomasz Skorski, Tomasz Śliwiński

**Affiliations:** 1 Laboratory of Medical Genetics Faculty of Biology and Environmental Protection, University of Lodz, 90-236 Lodz, Poland; 2 Department of Molecular Biology of Cancer, Medical University of Lodz, 92-215 Lodz, Poland; 3 Department of Flow Cytometry, Medical Center for Postgraduate Education, 01-813 Warsaw, Poland; 4 Department of Medical Biology, Medical University of Lodz, ul. Muszyńskiego 1, 90-151 Lodz, Poland; 5 Department of Medical Biochemistry, Medical University of Lodz, 92-216 Lodz, Poland; 6 Department of Surgical Oncology, Medical University of Lodz, 90-419 Lodz, Poland; 7 Department of Neurosurgery, Surgery of Spine and Peripheral Nerves, Medical University of Lodz, University Hospital WAM-CSW, 90-549 Lodz, Poland; 8 Fels Cancer Institute for Personalized Medicine, Lewis Katz School of Medicine, Temple University, Philadelphia, PA 19140, USA

**Keywords:** melanoma, HDACi, PARP1, valproic acid, alkylating agent, DNA damage

## Abstract

The inhibition of histone deacetylases (HDACs) holds promise as a potential anti-cancer therapy as histone and non-histone protein acetylation is frequently disrupted in cancer, leading to cancer initiation and progression. Additionally, the use of a histone deacetylase inhibitor (HDACi) such as the class I HDAC inhibitor—valproic acid (VPA) has been shown to enhance the effectiveness of DNA-damaging factors, such as cisplatin or radiation. In this study, we found that the use of VPA in combination with talazoparib (BMN-673—PARP1 inhibitor—PARPi) and/or Dacarbazine (DTIC—alkylating agent) resulted in an increased rate of DNA double strand breaks (DSBs) and reduced survival (while not affecting primary melanocytes) and the proliferation of melanoma cells. Furthermore, the pharmacological inhibition of class I HDACs sensitizes melanoma cells to apoptosis following exposure to DTIC and BMN-673. In addition, the inhibition of HDACs causes the sensitization of melanoma cells to DTIV and BMN-673 in melanoma xenografts in vivo. At the mRNA and protein level, the histone deacetylase inhibitor downregulated RAD51 and FANCD2. This study aims to demonstrate that combining an HDACi, alkylating agent and PARPi could potentially enhance the treatment of melanoma, which is commonly recognized as being among the most aggressive malignant tumors. The findings presented here point to a scenario in which HDACs, via enhancing the HR-dependent repair of DSBs created during the processing of DNA lesions, are essential nodes in the resistance of malignant melanoma cells to methylating agent-based therapies.

## 1. Introduction

The use of properly selected inhibitors of DNA double-stranded breaks (DSBs) repair proteins to induce cell death, mediated by the synthetic lethality (SL) phenomenon in tumor cells with a reduced activity of the main components of this type of repair, is a perspective approach to the application of personalized medicine for treating human solid tumors [1].

When looking at the five-year survival rate, melanoma proves to be an aggressive tumor, characterized by a low ten percent survival rate at the advanced stage of disease. Systemic therapy serves as the primary treatment approach for the majority of patients with metastatic melanoma, although surgery and radiation therapy also play a role. Although single-agent chemotherapy is generally well tolerated, it demonstrates limited efficacy, with response rates ranging from 5% to 20%. Combination chemotherapy and biochemotherapy have the potential to enhance the objective response rates; however, they do not confer a survival benefit and are linked to increased toxicity. Residual disease following combination treatment presents a significant challenge, as it can contribute to disease relapse, metastasis, and reduced overall survival rates. Strategies to address residual disease in this context are actively being explored to improve treatment outcomes and reduce the impact of residual disease in patients with metastatic melanoma [2,3].

The elimination efficacy of tumor cells can be enhanced by the administration of cytotoxic compounds used in standard chemotherapy, i.e., dacarbazine (DTIC) and temozolomide (TMZ) [4,5]. As DNA alkylating agents, they can induce the formation of ssDNA stretches. If these stretches of ssDNA are not bypassed by homologous recombination during DNA synthesis, DSBs will arise and trigger apoptosis in cancer. In cases of cancer cells without any defects in this repair system, it is possible to trigger such defects through the inhibition of histone deacetylases (HDACs), which reduces the activity of DSB repair key proteins, such as RAD51 or FANCD2 [1,6]. Recent studies also suggest that an oral chemotherapeutic agent, TMZ, used in the treatment of glioblastoma, triggers the activation of the DNA damage response (DDR) in resistant glioma cells, leading to the bypass of DNA damage and cell survival. The catalytic activity of a class I HDAC stimulates the expression of the E3 ubiquitin ligase RAD18. Furthermore, data have shown that RAD18 is part of the O6-methylguanine-induced DDR, as TMZ induces the formation of RAD18 foci at DNA damage sites. The downregulation of RAD18 through HDAC inhibition prevents DDR activation in glioma cells following exposure to TMZ [7].

Poly (ADP-ribose) polymerases (PARPs) are a group of enzymes involved in DNA repair pathways, particularly in base excision repair (BER) and single-strand break repair. However, PARPs also play a key role in the repair of DSBs through a pathway known as an alternative non-homologous end joining (al-NHEJ). PARP inhibitors (PARPis) exhibit a synthetic lethal effect in cells with deficiencies in HR repair, such as those with mutations in Breast Cancer gene 1 or 2 (BRAC1, BRAC2) as they rely heavily on the PARP-mediated alt-NHEJ pathway to repair DSBs. Numerous PARP inhibitors (Veliparib, Fluzoparib, Talazoparib, Olaparib, Rucaparib, Niraparib) have obtained approval by the USA Food and Drug Administration (FDA) and European Medicines Agency (EMA) for their application in the treatment of lung, breast, ovarian, pancreatic and prostate cancer. Nevertheless, akin to other targeted therapies, PARPi resistance frequently emerges, facilitated by diverse molecular mechanisms, despite their favorable tolerability and extensive clinical practice.

Histone deacetylases (HDACs) are a class of enzymes that play important roles in regulating gene expression by removing acetyl groups from histone proteins. This leads to a more compact chromatin structure, which can inhibit transcriptional activity. HDACs are divided into four classes based on their sequence homology and catalytic mechanism: Class I (HDAC1, HDAC2, HDAC3, and HDAC8), Class II (HDAC4, HDAC5, HDAC6, HDAC7, HDAC9, and HDAC10), and Class IV (HDAC11). The majority of these classes are zinc-dependent and function by using a catalytic zinc ion in their active site to deacetylate histones. However, Class III HDACs, also known as sirtuins (SIRT1-7), are NAD(+)-dependent and do not rely on zinc for their enzymatic activity [8]. Histone deacetylase inhibitors (HDACis) are a diverse group of drugs that modify the epigenome by changing the acetylation of not only histones but also other proteins not associated with histones [9]. HDACi drugs, particularly those that can inhibit all HDACs, also known as “pan HDACis”, have been found to reduce the expression of several DNA damage repair molecules [10]. This reduction in expression can occur through transcriptional downregulation of molecules such as BRCA-1, Checkpoint kinase 1 (CHK1), and RAD51, or through increasing the acetylation of HSP90 [11]. This increase in acetylation impairs the chaperoning function of HSP90, which leads to the decreased stability of DNA damage response (DDR) proteins [5]. Since Class I HDACs are often overexpressed in cancers and may contribute to those cancers’ response to therapy, we hypothesize that VPA—a histone deacetylase inhibitor, could sensitize melanomas to genotoxic agents such as dacarbazine (DTIC) administered alone or in combination with a PARP inhibitor.

## 2. Materials and Methods

### 2.1. In Vitro Cell Cultures

The patient-derived melanoma cell line DMBC11 was obtained from tumor specimens. The study was approved by the Ethical Commission of the Medical University of Lodz, and informed consent was obtained from all patients. Melanoma cells were cultured in a serum-free Stem Cell Medium (SCM) consisting of DMEM/F12 low osmolality medium (Lonza, Basel, Switzerland) in the presence of B-27 supplement (Gibco, Paisley, UK), growth factors (10 ng/mL bFGF and 20 ng/mL EGF; BD Biosciences, San Jose, CA, USA), insulin (10 μg/mL), heparin (1 ng/mL), and antibiotics (100 IU/mL penicillin, 100 μg/mL streptomycin, and 2 μg/mL fungizone). Cell cultures were maintained in low-adherent flasks (NUNC) at 37 °C in a humidified atmosphere containing 5% CO_2_ [12].

Normal Human Epidermal Melanocytes (NHEMs, Lonza) were cultured in Melanocyte Cell Basal Medium (MBM) (CC-3250, Lonza). The culture medium was supplemented with growth supplements containing CaCl_2_, hFGF-B, PMA, rh-Insulin, hydrocortisone, BPE, FBS (10%), gentamicin/amphotericin-B and endothelin (Lonza). Cells were maintained at 37 °C in a humidified atmosphere containing 5% CO_2_.

### 2.2. Drug Treatment

Melanoma cells and NHEMs were plated at a density of 1 × 10^5^ viable cells per well in a 12-well plate one day before drug treatment. Valproic acid sodium salt (VPA, Sigma-Aldrich) was dissolved to 100 mmol/L in PBS and stored at −80 °C after sterile filtration. Cells were pretreated with 1 mM valproic acid (VPA) for 168 h with a refreshment of VPA-containing medium every 48 h, followed by VPA removal before further treatment. After 168 h cells were cultured with 50 nM talazoparib (BMN-673) (MedChemExpress; Cat#HY-16106), 2 mM dacarbazine (DTIC) (Sigma Aldrich, Burlington, MA, USA). After 48 h, 1 mL of fresh medium containing drugs at appropriate concentrations was added to the cell culture for an additional 72 h of culturing.

### 2.3. Cell Viability

Melanoma cells were plated at a density of 1 × 10^5^ viable cells per well in a 24-well plate and pretreated with 1 mM valproic acid (VPA) for 168 h with a refreshment of VPA-containing medium every 48 h, followed by VPA removal before further treatment. After 168 h, the cells were cultured with 50 nM talazoparib (BMN-673) (MedChemExpress; Cat#HY-16106), and 2 mM dacarbazine (DTIC) (Sigma Aldrich, Burlington, MA, USA) used alone or in combination. A trypan blue exclusion assay was used to determine the viability of the cells after treatments with VPA, BMN-673, and DTIC. Cells were counted within 3 to 5 min of mixing with 0.4% trypan blue using light microscopy with a Neubauer hemocytometer. The experiments were carried out three times, in triplicate.

### 2.4. Cell Proliferation

Melanoma cells were incubated with compounds at the indicated concentrations for 120 h. Each well of the 12-well culture plates was coated with 350 μL bottom agar mixture (either SCM, 0.5% (*w*/*v*) agar, 5% FBS, or 0.5% (*w*/*v*) agar). After the bottom layer solidified, 350 μL of top agar medium mixture, containing 1 × 10^3^ cells was added (either SCM, 0.35% (*w*/*v*), 5% FBS, or 0.35% (*w*/*v*) agar). The plates were incubated at 37 °C in a humidified atmosphere containing 5% CO_2_. After 10 days of incubation, cell proliferation was determined using a clonogenic assay. For this purpose, the colonies were fixed and stained with 250 μL of 0.005% crystal violet for 1 h, and the spheres were counted under the microscope.

### 2.5. Apoptosis and Necrosis

FITC Annexin V staining precedes the loss of membrane integrity, which accompanies the latest stages of cell death resulting from either apoptotic or necrotic processes. Therefore, staining with FITC Annexin V is used in conjunction with a vital dye, such as propidium iodide (PI) or 7-Amino-Actinomycin (7-AAD), to allow the identification of early apoptotic cells (PI negative, FITC Annexin V positive). Viable cells with intact membranes exclude PI, whereas the membranes of dead and damaged cells are permeable to PI. An FITC Annexin V Apoptosis Detection Kit (BD Biosciences, Cat#556547) was used to quantitatively determine the percentage of cells within a population that were actively undergoing apoptosis, according to the manufacturer’s instructions. The cells were plated into a 24-well plate (1 × 10^5^ cells/well) and pretreated with 1 mM valproic acid (VPA) for 168 h with a refreshment of VPA-containing medium every 48 h, followed by VPA removal before further treatment. After 168 h, the cells were cultured with 50 nM talazoparib (BMN-673) (MedChemExpress; Cat#HY-16106), and 2 mM dacarbazine (DTIC) (Sigma Aldrich, Burlington, MA, USA). Following 24 h incubation with VPA, BMN-673 and DTIC used alone or in combination, the cells were washed twice with cold PBS and then resuspended in 1X Binding Buffer at a concentration of 1 × 10^5^ cells/mL. Next, 100 μL of the solution (1 × 10^4^ cells) was transferred to a 5 mL culture tube. Subsequently, 5 μL of FITC Annexin V and 5 μL of PI were added. The cells were gently vortexed and incubated for 15 min at room temperature (25 °C) in the dark. Finally, 400 μL of 1X Binding Buffer was added to each tube, and the samples were analyzed using flow cytometry within 1 h.

### 2.6. Histone γ-H2AX

The DMBC11 cell line was cultured at a density of 1 × 10^5^ cells per well. The H2AX Phosphorylation Assay Kit (Flow cytometry; Millipore, Cat#17-344) was used for the detection of the phosphorylated Histone H2AX levels. The assay was performed on cultured cells that were treated with agents inducing DNA damage or apoptosis (cells were pretreated with 1 mM VPA for 168 h with a refreshment of VPA-containing medium every 48 h, followed by VPA removal before further treatment). After 168 h, the cells were cultured with 50 nM BMN-673, and 2 mM DTIC), thereby promoting H2AX phosphorylation. Following treatment, the cells were fixed and permeabilized to facilitate staining and detection. The presence of Histone H2AX phosphorylated at serine 139 was detected using the FITC-conjugated anti-phospho-Histone H2AX antibody. Flow cytometry was employed to quantify the number of cells exhibiting positive staining for phosphorylated histone H2AX.

### 2.7. RNA Isolation, cDNA Synthesis and Real-Time PCR

RNA was extracted from the cultured DMBC11 pellet 2.5 × 10^6^ using a total RNA isolation kit (A&A Biotechnology; Cat#031-100). Following that, the RNA was transcribed into complementary DNA using SuperScript II Reverse Transcriptase from Invitrogen Life Technologies. For the quantitative reverse transcription PCR (qRT-PCR), TaqMan Real-Time PCR Master Mix from Life Technologies was utilized, and the qPCR reactions were conducted on an Agilent Technologies Stratagene Mx3000P system with MxPro software. The expression levels of seven genes whose products are involved in the DNA double-strand break repair pathways (*RAD51*, *RAD51D*, *FANCD2*, *BRCA1*, *BRAC2*, *PALB2*, *PARP1*) were analyzed using TaqMan probes (Life Technologies CA, USA). The reference gene used was 18S RNA (Life Technologies, CA, USA). The qPCR cycling parameters were 95 °C for 10 min, 30 cycles of 95 °C for 15 s, and 60 °C for 60 s.

### 2.8. Preparation of Protein Extracts and Western Blot Analysis

Protein extraction was performed by washing the cell pellet with ice-cold PBS. Next, 1 mL of ice-cold RIPA lysis buffer (Sigma) and a protease inhibitor cocktail (Thermo Scientific, Rockford, IL, USA) were added and incubated on ice for 30 min. Then, 30 µg of cell lysates were resolved on 4–20% ExpressPluS PAGE Gel (GenScript, Piscataway, NJ, USA) following concentration measurements. The eBlot Protein Transfer device (GenScript, Piscataway, NJ, USA) was used to transfer the proteins onto a PDVDF Transfer Membrane (Thermo Scientific, Rockford, IL, USA). The membranes were washed and incubated for 1 h with a secondary anti-mouse antibody conjugated with HRP (Cell Signaling Technology, Danvers, MA, USA). Pierce ECL Western blotting Substrate (Thermo Scientific, Rockford, IL, USA) and BioRad Universal Hood II with a Chemiluminescence System (BioRad, Hercules, CA, USA) were used to visualize the result. Antibodies: Recombinant Anti-FANCD2 antibody [EPR2302] (abcam—ab108928) 1/1000; Recombinant Anti-Rad51D antibody [EPR16205] (abcam—ab202063) 1/1000; Recombinant Anti-β Actin antibody [EPR21241] (abcam—ab213262) 1 µg/mL; Recombinant Anti-Rad51D antibody [EPR16205] (ab202063) 1/1000.

### 2.9. Neutral Comet Assay

A comet assay was performed according to the protocol used in the previous research [5] on cells cultured for 48 h with either drugs or the vehicle and 168 h of pretreatment with VPA. Fifty comet images were randomly selected for each treatment variant and the percentage of DNA in the tail (% tail DNA) was measured. The mean value for this parameter was taken as an index of DSBs in the given sample.

### 2.10. In Vivo Experiments

NOD SCID γ (NSG) mice (10- to 12-week-old males and females) were housed in a sterile environment and allowed free access to food and water. The studies with animal experiments were approved by the local Ethical Committee and performed according to Polish federal law and guidelines for the protection of animals. DMBC11 human melanoma xenografts were initiated by injecting (under the right scapula) 2 × 10^6^ cells previously suspended in Matrigel. Three animals were assigned to the eight different groups. The groups that received valproic acid pretreatment were treated as follow. Six days before the BMN-673 and DTIC injection, the mice were injected intraperitoneally once daily with 500 mg/kg VPA (diluted in PBS). Following pretreatment with VPA, the mice were treated with BMN-673 (35 mg/kg bodyweight, diluted in PBS), DTIC (8 mg/kg bodyweight every second day, diluted in PBS) or BMN-673 with DTIC (same dosing as monotherapy) for 24 days. At the end of the experiment, tumors were collected and weighed.

### 2.11. Statistical Analysis

The data were accessed in three independent experiments and presented as the mean ± SD. The student’s *t*-test was performed between the various treatment regimens using Prism 7 (GraphPad Software, La Jolla, CA, USA). Differences were considered statistically significant where *p* < 0.05. The synergistic effect of drugs was studied using the response additivity approach.

## 3. Results

### 3.1. VPA Increase the Cytotoxic Effect Induced by BMN-673 and DTIC

**Figure 1:**
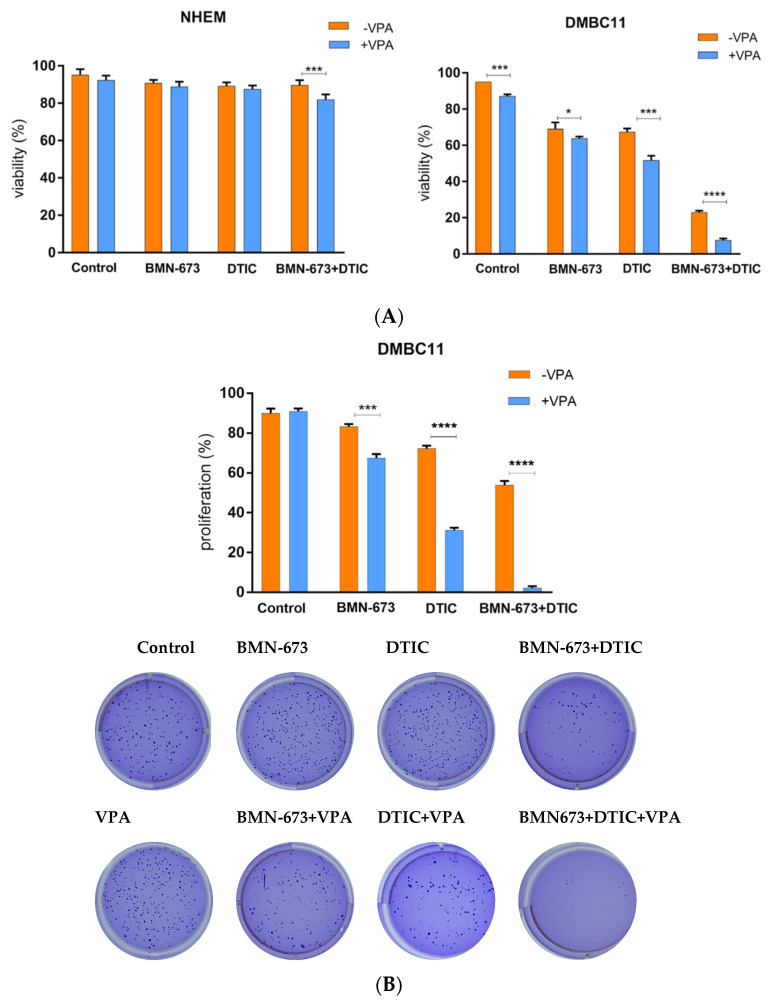

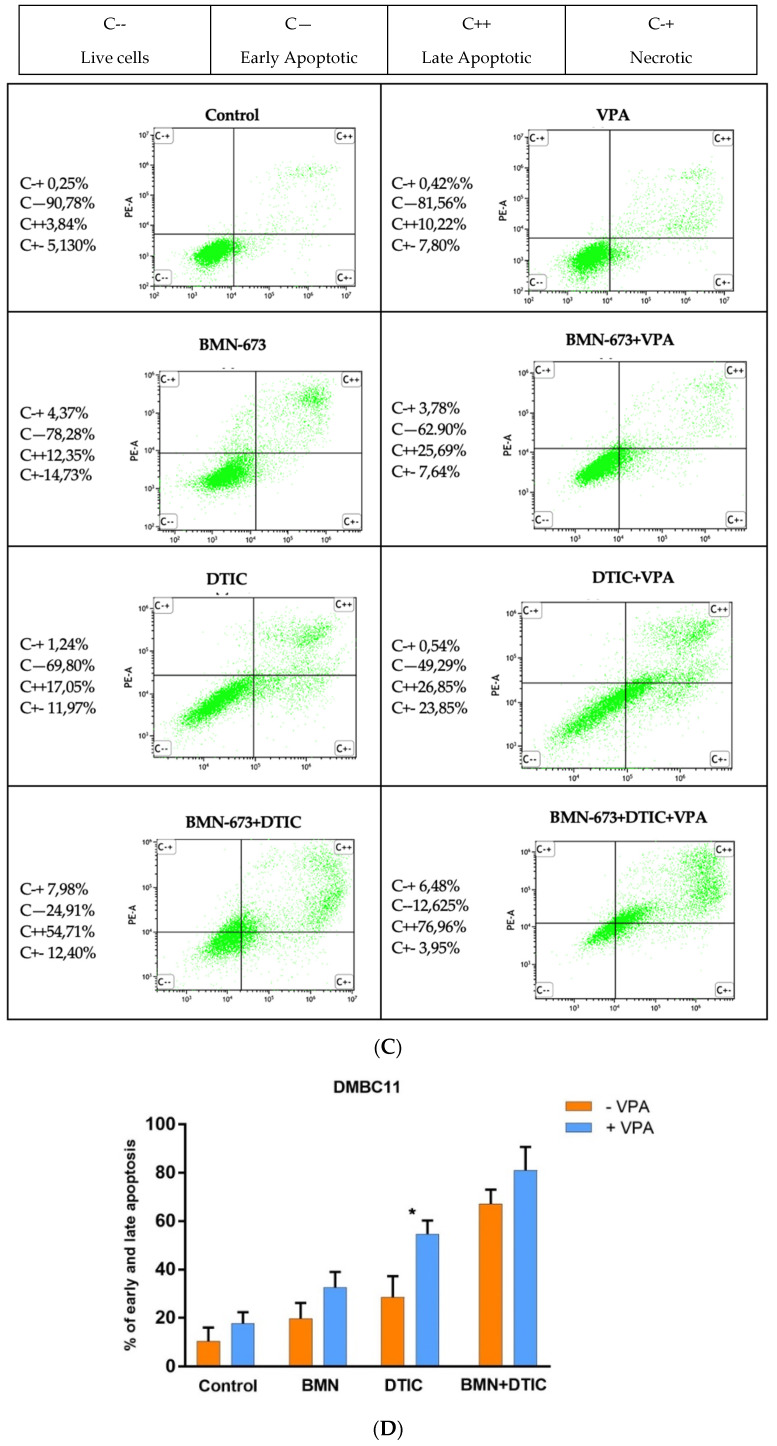
VPA increases the cytotoxic effect of the DTIC alkylating agent and BMN-673 PARP1 inhibitor. DMBC11 cells were pretreated with VPA (1 mmol/L) for 168 h and then treated or not with BMN-673 (10 μM) and DTIC (2 mM). (**A**) Cell viability was evaluated with a trypan blue assay after 72 h of treatment. (**B**) Cell proliferation was evaluated with a clonogenic assay after 72 h of treatment. (**C**) Representative histograms of the apoptosis/necrosis analysis of melanoma cells after 24 h of treatment with DTIC and BMN-673 (and pretreatment with VPA). (**D**) The percentage of DMBC11 melanoma cells in early and late apoptosis. At least three independent experiments were performed, and the results are shown as the mean ± standard deviation (SD). * *p* ≤ 0.05, *** *p* ≤ 0.001, **** *p*-value ≤ 0.0001 compared with the control group.

To assess how the tested compounds affected the number of viable cells, we used trypan blue to measure plasma membrane integrity (Figure 1A). Combining VPA with DTIC and/or BMN-673 resulted in a synergistic reduction in DMBC11 cell survival, while the normal human epidermal melanocytes (NHEMs) were not affected by the treatments. To evaluate the impact of the compounds on cell proliferation, we used a clonogenic assay to obtain distinct colonies. When used alone, DTIC and BMN-673 reduced the number of colonies (Figure 1B). However, when the drugs were used in combination with VPA, this resulted in increased clonogenic inhibition efficiency. Treatment with a combination of VPA, BMN-367 and DTIC led to more significant changes (Figure 1C) than those observed with single drug treatments. The number of apoptotic cells significantly increased after treatment with the VPA, BMN-673, and DTIC combination compared to what was reported for monotherapy.

### 3.2. VPA, DTIC and BMN673, Used Alone or in Combination, Increase the Number of DSBs in Melanoma Cells

**Figure 2:**
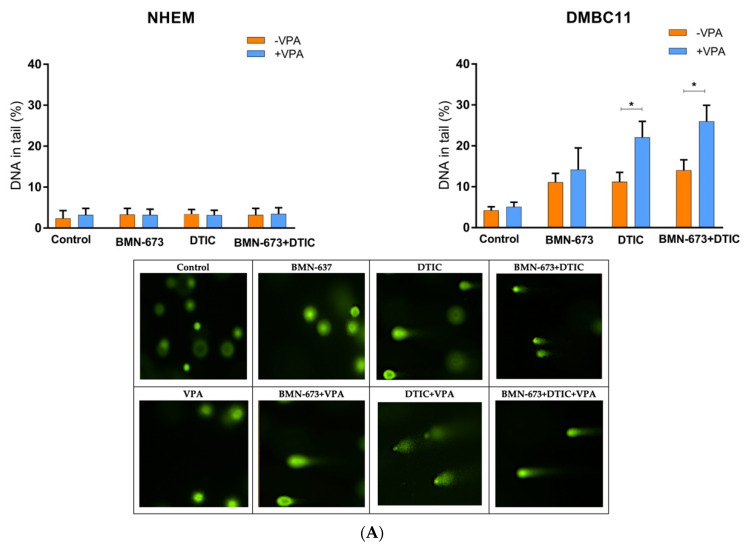

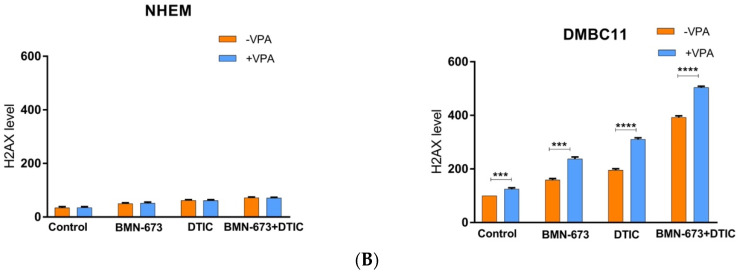
BMN673 and/or DTIC, VPA induced DSBs in melanoma cell lines (DMBC11). Cells were treated with 80 nM BMN-673 and/or 2 mM DTIC and VPA for 48 h (comet assay) and 120 hrs (γ-H2AX). (**A**) The mean percentage ± SD of DNA in the tails of comets in neutral conditions acquired from the 50 cells/group from 3 experiments. The characteristic tails of comets with damaged DNA are shown under a fluorescence microscope after electrophoresis and 4′,6-diamidino-2-phenylindole (DAPI) gel staining. (**B**) The mean values ± SD of γ-H2AX were calculated from 3 experiments performed in triplicate. The characteristic tails of comets with damaged DNA are shown under a fluorescence microscope after electrophoresis and 4′,6-diamidino-2-phenylindole (DAPI) gel staining. * *p*-value ≤ 0.05; *** *p*-value ≤ 0.001; **** *p*-value ≤ 0.0001 in comparison with the control.

In normal melanocytes, treatment did not alter the level of γ-H2AX, which marks DSBs. However, the DMBC11 cells exhibited increased levels of γ-H2AX compared to melanocytes (Figure 2A). Additionally, the combined treatment with VPA approximately doubled the level of phosphorylated γ-H2AX in the DMBC11 cells compared to treatment with either drug alone.

The neutral comet assay was used to measure the ability of VPA and/or DTIC and BMN673 to induce DSBs. DMBC11 cells treated with individual drugs exhibited an increased intensity of DNA tails compared to melanocytes, indicating the accumulation of DSBs (Figure 2B). Moreover, the combination of VPA, DTIC, and BMN673 caused more DSBs than individual drugs.

### 3.3. Inhibition of HDAC1 Downregulates FANCD2 and RAD51

**Figure 3:**
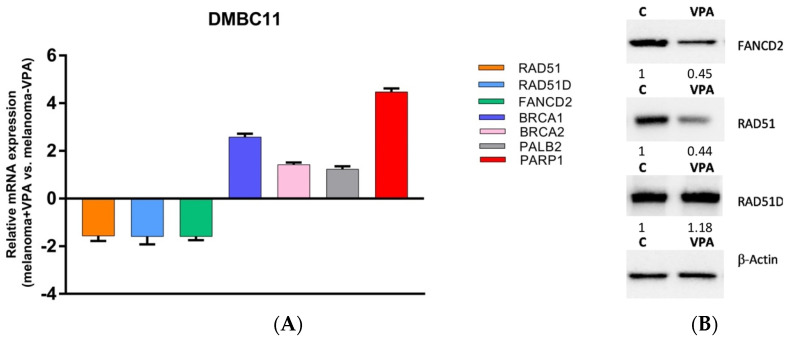
VPA and downregulated RAD51 and FANCD2 in melanoma cells. DMBC11 cells were pretreated with VPA (10 mM) for 178 h. (**A**) Real-time PCR results showing the down and upregulation of the listed genes. (**B**) Western blot analysis of the RAD51, RAD51D and FANCD2 protein levels. β-Actin served as a loading control and the relative expression (R.E.) levels are indicated.

To investigate the mechanism by which class I HDAC inhibition enhances sensitivity to dacarbazine and talazoparib, we conducted a real-time PCR array to monitor changes in mRNA expression in DMBC11 melanoma cells following the inhibition of class I HDACs using VPA (Figure 3A). The subjects of our interest were seven genes involved in the double strand break repair pathway (RAD51, RAD51D, FANCD2, BRCA1, BRCA2, PALB2, PARP1). We observed changes in the mRNA expression profile of RAD51, RAD51D, and FANCD2.

We then confirmed whether the changes in gene expression were reflected at the protein level by analyzing the influence of VPA on the RAD51, FANCD2, and RAD51D protein levels (Figure 3B). The results showed that only the RAD51 and FANCD2 protein levels decreased following HDAC inhibition.

### 3.4. VPA in Combination with BMN673 and DTIC Reduces Melanoma Growth in NSG Mice

**Figure 4:**
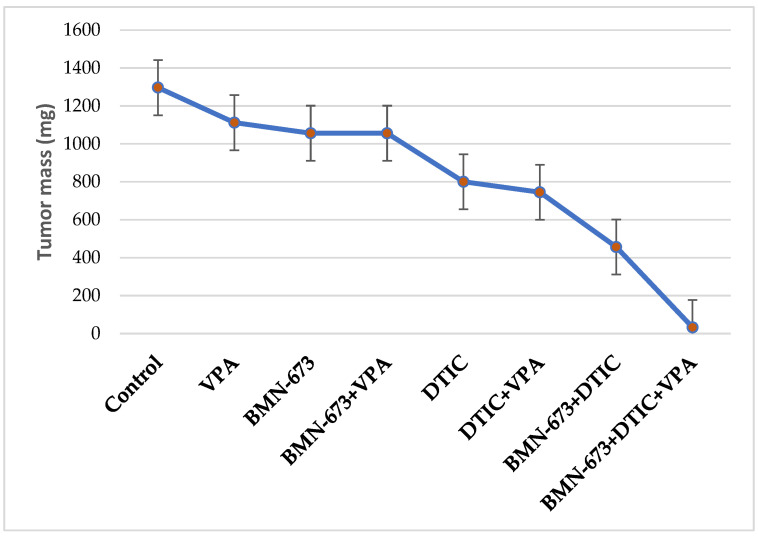
Combination of VPA, DTIC and BMN673 reduced the growth of human melanoma in immunodeficient mice. NAG mice were injected s.c. with DMBC11 melanoma cells followed by treatment with BMN673 (35 mg/kg twice a day), DTIC (8 mg/kg every second day), or BMN673 + DTIC. Data represent the mean ± SD of the tumor mass from 2 independent experiments.

In NSG mice, suboptimal doses of BMN673, VPA, and DTIC did not show any significant reduction in the growth of DMBC11 cells (Figure 4). However, a modest but statistically significant anti-melanoma effect was observed when BMN673 was combined with DTIC, and when BMN673, DTIC, and VPA were used together. It is worth noting that a more potent effect could potentially be achieved through optimization of the treatment protocol.

## 4. Discussion

Recent years have seen significant progress in the development and expanded application of PARP and HDAC inhibitors due to a growing interest in their potential for cancer therapy [13]. Metastatic melanomas are resistant to therapy for a variety of reasons, including their ability to bypass cell-cycle checkpoints [14], express insufficient amounts of critical apoptosis proteins [15], maintain p53 wild-type status, which allows them to upregulate DNA repair genes (DDB2, XPC) [16], and express an oncogenic form of BRAF that gives them an advantage in growth [17]. This may be the reason why melanoma patients have such a poor prognosis.

Before examining whether class I HDACs play a role in melanomas’ susceptibility to an alkylating agent and PARP inhibitor, we demonstrated that these HDACs can be suppressed. In order to achieve this, the class I HDAC inhibitor VPA [15], which was utilized, was proven to be functional in melanoma cells. Having established the experimental system, we demonstrated that the inhibition of class I HDACs sensitized melanoma cells to apoptosis induced by dacarbazine. Furthermore, a panel of cell lines in vitro and a melanoma xenograft model in vivo corroborated the protective effect of class I HDAC activity in melanomas.

HDACs have a crucial role in regulating proteins and gene expression. As a result, HDACs can potentially influence the expression of any gene, necessitating the use of screening methodologies to identify the underlying factor responsible for the observed phenotype. This is accomplished by modifying the acetylation of both histones and non-histone molecules [18,19].

In this study, we have identified the combination of an HDAC inhibitor, PARP inhibitor and alkylating agent as a potentially effective therapeutic approach against melanoma cells. Specifically, we found that VPA enhanced the cytotoxicity of DTIC and BMN-673. This increase in cytotoxicity was associated with a stronger induction of DNA damage in cells treated with the HDACi/BMN-673/DTIC combination, likely due to the downregulation of FANCD2 and RAD51 by VPA. The observed stronger cytotoxicity of the combination of an HDACi, PARPi, and alkylating agent may be attributed to the fact that one of the mechanisms underlying tumor resistance to PARP inhibitors is the activation of the HR DNA repair pathway. Romeo et al. obtained similar results, where the HDAC inhibitor VPA, and trichostatin A (TSA), disrupted the interplay between mutp53 and HSP70, resulting in the downregulation of CHK1 and RAD51, sensitizing pancreatic cancer cells to the AZD2461 PARP inhibitor [20].

In this study, a real-time PCR was utilized to investigate the effects of VPA on selected genes’ expression, which revealed that VPA impacts genes involved in DNA repair (especially HR). Based on this finding, it is inferred that VPA sensitizes melanoma cells to dacarbazine and talazoparib by either improving apoptosis fidelity or reducing DNA repair effectiveness. To confirm these hypotheses, changes in gene expression were validated using Western blot analysis, revealing RAD51 and FANCD2 as the two proteins affected by VPA. By using an HDAC inhibitor to target specific HDACs, HDAC2 was identified as the class I HDAC responsible for the regulation of RAD51 [1]. Since RAD51 and FANCD2 are critical components of HR, downregulation of these proteins could be responsible for the observed sensitization. Krumm et al. obtained similar results by using various HDAC inhibitors and siRNA to target specific HDACs, identifying class I HDAC as being responsible for regulating RAD51. The role of DNA repair in the observed sensitization to temozolomide was further supported by the results obtained in melanoma cells exposed to fotemustine (alkylating agent) and ionizing radiation (IR) after the inhibition of class I HDAC. Although temozolomide, fotemustine, and IR induce different types of DNA damage, only HR is involved in the repair of all these genotoxins. Our observation of the cells treated with the monotherapy and polytherapy was consistent with previously described studies [1,5,21,22]. 

The findings presented here point to a scenario in which HDACs, via enhancing the HR-dependent repair of DSBs created during the processing of DNA lesions, are essential nodes in the resistance of malignant melanoma cells to methylating agent-based therapies. This provides a solid foundation for the targeting of these HDACs during genotoxic agent-based therapy for malignant melanoma and, perhaps, other tumor types as well.

## 5. Conclusions

This study suggests that VPA synergizes with the PARP inhibitor and alkylating agent to reduce the survival of melanoma cells, due to stronger DNA damage being induced by this combination treatment, while not affecting melanocytes.

Interestingly, the interconnection between the effects induced by an HDACi has never been highlighted before in cancer cells. The findings of this study encourage the use of an HDACi, especially one that inhibits class I HDACs, in combination with a PARP inhibitor in the treatment of melanoma cancer.

## Data Availability

The data presented in this study are available upon request.
